# Mitochondrial dysfunction induced by bedaquiline as an anti-*Toxoplasma* alternative

**DOI:** 10.1186/s13567-023-01252-z

**Published:** 2023-12-19

**Authors:** Yuehong Shi, Yucong Jiang, Haolong Qiu, Dandan Hu, Xingju Song

**Affiliations:** 1 College of Animal Science and Technology, Guangxi University, Nanning, 530004 China; 2 Guangxi Zhuang Autonomous Region Engineering Research Center of Veterinary Biologics, Nanning, 530004 China; 3 Guangxi Key Laboratory of Animal Breeding, Disease Control and Prevention, Nanning, 530004 China

**Keywords:** *Toxoplasma gondii*, bedaquiline, mitochondria, autophagy, RNA-seq

## Abstract

*Toxoplasma gondii* is a zoonotic parasite that infects one-third of the world’s population and nearly all warm-blooded animals. Due to the complexity of *T. gondii’s* life cycle, available treatment options have limited efficacy. Thus, there is an urgent need to develop new compounds or repurpose existing drugs with potent anti-*Toxoplasma* activity. This study demonstrates that bedaquiline (BDQ), an FDA-approved diarylquinoline antimycobacterial drug for the treatment of tuberculosis, potently inhibits the tachyzoites of *T. gondii*. At a safe concentration, BDQ displayed a dose-dependent inhibition on *T. gondii* growth with a half-maximal effective concentration (EC_50_) of 4.95 μM. Treatment with BDQ significantly suppressed the proliferation of *T. gondii* tachyzoites in the host cell, while the invasion ability of the parasite was not affected. BDQ incubation shrunk the mitochondrial structure and decreased the mitochondrial membrane potential and ATP level of *T. gondii* parasites. In addition, BDQ induced elevated ROS and led to autophagy in the parasite. By transcriptomic analysis, we found that oxidative phosphorylation pathway genes were significantly disturbed by BDQ-treated parasites. More importantly, BDQ significantly reduces brain cysts for the chronically infected mice. These results suggest that BDQ has potent anti-*T. gondii* activity and may impair its mitochondrial function by affecting proton transport. This study provides bedaquiline as a potential alternative drug for the treatment of toxoplasmosis, and our findings may facilitate the development of new effective drugs for the treatment of toxoplasmosis.

**Supplementary Information:**

The online version contains supplementary material available at 10.1186/s13567-023-01252-z.

## Introduction

*Toxoplasma gondii*, an intracellular parasitic protozoan of the phylum Apicomplexa, is a medically and veterinary important pathogen that can infect humans and almost all warm-blooded animals worldwide [1]. *T. gondii* can lead to serious secondary infections in immunocompromised populations and reproductive disorders in pregnant women. It is estimated that about one-third of the global population is infected with *T. gondii*. The most effective clinical treatment for toxoplasmosis is currently a combined therapy of sulfadiazine and pyrimethamine [2], which blocks the parasite’s folic acid metabolism*.* However, this procedure is ineffective against chronic infections and is accompanied by relatively severe side effects, including suppression of bone marrow [3] and teratogenic effects in early pregnancy [4]. In addition, other alternative drugs, such as spiramycin [5], azithromycin [6], and atovaquone [7], have shown limited effectiveness in clearing *T. gondii* and are not as effective as conventional therapy. Therefore, the search for more efficient and safer drugs for the treatment of toxoplasmosis remains an urgent issue.

The respiratory chain is a key pathway for the production of ATP by Apicomplexans and is considered as a possible drug target [8]. The mitochondrial ATP synthase (also known as complex V) of *T. gondii* is a key enzyme to produce ATP by oxidative phosphorylation, which is essential for maintaining the division and proliferation of the parasite [9, 10]. Mitochondrial ATP synthase is a multi-subunit complex consisting of two parts, F1 and F_O_ [11, 12]. Recent studies have shown that the sequence and structure of the F_O_ subunit of *T. gondii* ATP synthase are highly different from those of the host cell. These specific subunits interact to form a cristae-embedded ATP synthase hexamer arranged in a pentagonal pyramidal structure, which is different from the conventional ATP synthase structure of the host cell [13]. Therefore, the F_O_ subunit of ATP synthase may be a novel and promising drug target for *T. gondii*. Bedaquiline (BDQ) is an FDA-approved antimycobacterial drug for the treatment of multidrug-resistant tuberculosis. BDQ is a diarylquinoline drug that affects the activity of the ATP synthase proton pump [14, 15] and causes blockage of ATP synthesis, which results in interruption of the ATP energy supply in *Mycobacterium tuberculosis* to exert antibacterial and bactericidal effects. Recently, BDQ has been shown to be effective in combatting malaria by delaying the invasion of *Plasmodium* merozoites by inhibiting the formation of the parasite’s inner membrane complex (IMC) and increasing its ROS level [16].

In this study, the in vitro and in vivo anti-*Toxoplasma* activity of BDQ was tested. The results show potent inhibitory activity with low cytotoxicity in vitro. BDQ treatment resulted in destruction of mitochondrial structure and function of *T. gondii* tachyzoites and increased ROS levels in the parasite. To learn more about the mode of action of BDQ, the transcriptional profile of BDQ-exposed *T. gondii* was characterized. Our study suggests that BDQ has potent anti-*Toxoplasma* activity and also offers the possibility of repurposing BDQ to combat toxoplasmosis.

## Materials and methods

### Culture of cells and parasites

HFF (Human foreskin fibroblasts) and Vero (African green monkey kidney fibroblast) cells were purchased from the American Type Culture Collection (Manassas, VA, USA) and cultured in Dulbecco modified Eagle medium (DMEM) supplemented with 100 IU/mL penicillin and 0.1 mg/mL streptomycin (Beyotime, China), along with 10% fetal bovine serum (FBS). *T. gondii* RH strain expressing luciferase (TgRH-Luc) [17] was used to determine the growth of the parasite, as indicated by reduced luciferase activity. Parasites were maintained in cells containing 2% FBS at 37 °C and 5% CO_2_. Bedaquiline (BDQ) stock solution (MedChemExpress, Shanghai, China) was dissolved in 100% dimethyl sulfoxide (DMSO) and filtered using a 0.22-micron filter.

### Cytotoxicity test

The cytotoxicity of BDQ to Vero cells was evaluated by a CCK-8 assay. Vero cells were cultured in 96-well plates (5000 cells/well) for 24 h in medium containing 8% FBS and incubated at 37 °C and 5% CO_2_. Cells were exposed to the compounds at final concentrations of 1, 2.5, 5, 10, 20, 40, 80 and 160 μM. Blank control wells containing medium without cells were also prepared for background-deducted fluorescence measurements. After removal from the medium, cell viability was determined using CCK-8 reagent according to the manufacturer’s instructions. Absorbance was measured at 450 nm using a Tecan Infinite M200 Pro (Tecan, UK). Cytotoxicity was calculated using the following formula: % viability = [(OD_BDQ_—OD_blank_) / (OD_DMSO_—OD_blank_)] × 100. The final concentration of up to 1% DMSO in the culture medium did not affect cell proliferation. Graphical presentations and statistical analyses (unpaired *t*-test) were completed using GraphPad Prism 9 (San Diego, CA, USA).

### In vitro drug inhibition assay

Vero cells were seeded onto 96-well clear-bottom plates and incubated at 37 °C and 5% CO_2_. Cells were then infected with 3.0 × 10^5^ TgRH-Luc per well. Thereafter, the wells infected with *T. gondii* were exposed to different concentrations of BDQ (1 to 50 μM). DMSO (0.1%) was used as a negative control. After 24 h of treatment, parasite viability was measured using a Firefly Luciferase Reporter Gene Assay Kit (Beyotime, China). The relative luminescence unit (RLU) of each well was measured using a Tecan Infinite M200 Pro reader (Tecan, UK). The percentage of growth inhibition relative to the luminescence values of the negative control was calculated using the following equation: %inhibition = (RLU_DMSO_-RLU_treatment_) / RLU_DMSO_ × 100. Three replicates are shown for each condition. The half-maximal effective concentration (EC_50_) of BDQ and 95% confidence interval (CI) were calculated using the regression equation for the slope of the log (inhibitor) and response variables (four parameters) in GraphPad Prism 9.

### Plaque assay

HFF growing in 12-well plates were infected with 150 freshly harvested tachyzoites and incubated for 8 days without disturbance. The experimental group was incubated under the same conditions at approximately 4 times the EC_50_ concentration of the drug (20 μM). Thereafter, infected HFF were fixed by 4% paraformaldehyde and observed by 0.2% crystal violet solution staining. Plaque area was counted by pixels using Photoshop C6S software (Adobe, USA), and data were compiled from three independent experiments.

### Immunofluorescence assay

Freshly harvested tachyzoites were inoculated on HFF monolayers grown in 12-well plates. Infected cells were fixed in 4% paraformaldehyde. They were then followed with 0.25% Triton X-100 in PBS for 30 min and blocked with 3% bovine serum albumin (BSA) for 30 min. Subsequently, they were incubated with primary rabbit anti-TgGAP45 (1:300), mouse anti-FLAG (1:300, Sigma, USA), rabbit anti-TgIMC1 (an inner membrane complex marker, from Prof. Liu at China Agricultural University) (1:300), and mouse anti-TgACP (acyl carrier protein, a marker for apicoplast, from Prof. Liu at China Agricultural University) (1:300) for 1 h, washed three times with PBS and then incubated with secondary FITC- or Cy3-conjugated antibodies for 1 h. Cell nuclei were stained with Hoechst 33258 (Sigma, USA). The images were obtained using a Zeiss Fluorescence Microscopy system (Zeiss, Germany).

### Intracellular and extracellular inhibition assays

For the extracellular inhibition assay, Vero cells were seeded onto 96-well clear-bottom plates and incubated at 37 °C and 5% CO_2_. Extracellular TgRH -Luc tachyzoites were exposed to BDQ for 4 h and then allowed to infect Vero for 24 h after several washes to remove the compound. Cells and parasites were then lysed, and RLU were measured as described above. The results of three independent experiments are shown, with each condition repeated three times. For the intracellular inhibition assay, Vero cells in 96 wells were infected with 3 × 10^5^ TgRH-Luc parasites for 2 h, and subsequently co-cultured with BDQ (10 μM) for an additional 24 h at 37 °C with 5% CO_2_. Cells and parasites were then lysed and RLU were measured. Three independent experiments were performed.

### Intracellular replication and invasion assay

The intracellular replication effect of BDQ on *T. gondii* was observed by immunofluorescence microscopy. Vero cells in 12-well plates were infected with 3 × 10^5^ fresh RH tachyzoites per well and incubated for 2 h. The cell surface was then washed twice with PBS to remove extracellular tachyzoites, and then incubated with 10 μM BDQ. After 20 h of treatment, cells were fixed with 4% paraformaldehyde, followed by immunofluorescence staining using rabbit anti-TgGAP45 antibodies and Hoechst. The number of parasites per strain was determined by counting at least 100 vacuoles using fluorescence microscopy. For the invasion assay, the percentage of invasion was shown as the number of vacuoles per host cell. DMSO was used as a vehicle control. Three independent experiments were carried out.

### ROS assay

Tachyzoites in Vero cells were treated with different concentrations of BDQ (0, 5, 10, or 20 μM) or DMSO for 16 h or 24 h. Tachyzoites treated with hydrogen peroxide (H_2_O_2_, 200 μM) were used as a positive control. Fresh tachyzoites (approximately 1 × 10^6^ /group) were then extracted and incubated with 10 µM H2DCFDA (DCFH-DA, 2’,7’-Dichlorodihydrofluorescein diacetate) probe for 20 min at 37 °C. After washing 3 times with serum-free DMEM, fluorescence intensities of the wells were measured at 488 nm excitation and 525 nm emission using a fluorescence microplate reader (Tecan, Infinite M200 PRO, Männedorf, Switzerland) [18]. The final value of ROS for each group was expressed as RLU/parasite.

### ATP level determination

After incubation with different concentrations of BDQ (0, 5, 10 and 20 µM) at 37 °C for 4 h, *T. gondii* tachyzoites (2 × 10^6^ per experimental group) were collected and immediately lysed on ice with 200 μL of lysis buffer. The lysates were subsequently centrifuged at 12 000 × *g* for 5 min, and 20 μL of the supernatants were added to the wells of an opaque-walled 96-well microplate, each well containing 100 μL of ATP detection working dilution (Beyotime, Shanghai, China). The luminescence of each well was detected using a multi-labeled fluorescence microplate reader (Tecan, Infinite M200 PRO, Männedorf, Switzerland). ATP levels were calculated based on an ATP standard curve, which was established by gradient dilution of the standard ATP sample supplied with the kit. Three independent experiments were assessed in triplicate.

### Detection of mitochondrial membrane potential (ΔΨm)

Changes in mitochondrial membrane potential of extracellular tachyzoites treated with BDQ were evaluated using the JC-10 assay kit (Biosharp, Shanghai, China) according to the manufacturer’s instructions. Briefly, fresh tachyzoites (2 × 10^6^) were treated with different concentrations of BDQ (0, 5, 10 and 20 μM) for 4 h at 37 °C. Subsequently, tachyzoites were incubated in 0.5 mL of staining solution containing the potentiometric probe JC-10 for 20 min at 37 °C in the dark. The samples were then rinsed twice with buffer and resuspended in JC-10 staining buffer. Finally, the FI signals of JC-10 (JC-10 Monomer wavelength of Ex = 490 nm / Em = 530 nm; JC-10 Polymer wavelength of Ex = 525 nm/Em = 590 nm) were detected using a fluorescence microplate reader (Tecan, Infinite M200 PRO, Männedorf, Switzerland). The ratio of fluorescence values of J-aggregates to those of monomers was calculated using the following formula: RLU _J-aggregates_ / RLU _J-Monomers_. Images were also taken at each treatment dose and control using a Zeiss Fluorescence Microscopy system (Zeiss, Germany). Carbonyl cyanide 3-chlorophenylhydrazone (CCCP), a protonophore that leads to ΔΨm depletion, was used as a positive control.

### Detection of parasite autophagy

As previously reported, parasite autophagy was detected by staining autophagic vacuoles [19]. Briefly, tachyzoites in Vero cells were treated with BDQ (0, 5, 10 and 20 μM) at 37 °C for 16 h. Freshly-harvested tachyzoites (1.5 × 10^6^) were suspended in monodansylcadaverine (MDC) solution for 30 min at 37 °C in the dark. After washing three times with the assay buffer in the MDC staining assay kit (Beyotime, Shanghai, China), fluorescence intensities of the samples were measured at excitation 335 nm and emission 512 nm using a fluorescence microplate reader (Tecan, Infinite M200 PRO, Männedorf, Switzerland), and the experiment was repeated three times.

### In vivo assay

Acute and chronic toxoplasmosis was studied in vivo by using six-week-old female Kunming mice. To study acute infection, ten mice were injected intraperitoneally (i.p.) with 100 ME49 tachyzoites or 1000 Pru tachyzoites, respectively. After 24 h of infection, BDQ (50 mg/kg b. wt.) were orally administered in vehicle solution (0.05% (Hydroxypropyl) methylcellulose with 0.2% Tween 80 in Normal saline [16]. The mice were humanely euthanized by cervical dislocation after anesthetization by subcutaneous injection of atropine (0.02 mg/kg) when they lost 20% body weight. The mice that remained healthy after infection were raised to the end of their lives. To establish chronic infection, mice were inoculated with 500 Pru tachyzoites. After 21 days post-infection, chronically infected mice were randomly grouped (5 mice/group) and administered with BDQ or vehicle (i.p.) for another 21 days.

To determine cyst burdens, tissue cysts in the brains were quantified by staining with Dolichos Biflorus Agglutinin (DBA; RL-1032, Vector Laboratories, Burlingame, CA, USA) that was labeled with FITC, and microscopic examination. Briefly, brain homogenate (100 µL) was fixed with 100 µL of paraformaldehyde containing 0.2% Trition-100 at 4 °C for 20 min. After fixation, the precipitate was centrifugated twice at 1000 *g* for 10 min and suspended with DMEM containing 10% FBS. Immediately, the precipitate was suspended with 100L of DMEM containing 10% FBS and stained with 1 μL of FITC-labeled DBA at room temperature and protected from light for 1 h. After several washes, the precipitate was suspended with 100 µL DMEM, and 10 µL drops were placed on slides (4 replicates per brain sample), and the cysts were counted under a fluorescence microscope. The number of cysts in each brain was calculated.

### Electron microscopy

Ultrastructural analysis was performed as previously reported [17]. Briefly, intracellular tachyzoites were treated with BDQ or DMSO for 15 h, then were collected and fixed with fresh 2.5% glutaraldehyde (ServiceBio, Wuhan, China) for 30 min at 4 °C. Fixed samples were treated with 1% OsO_4_ in 0.1 M Phosphate Buffer (pH 7.4) for 2 h at RT. After dehydration with a serial gradient concentration of ethanol (30%-100%), samples were embedded in pure resin and polymerized for 48 h at 50 °C. The ultrathin Sects. (70 nm thick) were sectioned with a microtome (Leica UC7, Leica, Germany), and were fixed onto the 150 meshes cuprum grids with formvar film. Finally, the ultrathin sections were stained with 2% uranyl acetate and lead citrate, and observed under a Transmission Electron Microscope (HT7800, Hitachi, Japan).

### CRISPR mediated endogenous gene tagging

The ToxoDB database was used to design the gRNA in the corresponding gene-specific CRISPR–Cas9 plasmids. The construction of CRISPR/Cas9 plasmids was performed as previously described [20]. Briefly, the Cas9 upstream and downstream fragments containing gRNA sequences were amplified and ligated using a seamless cloning kit (Vazyme Biotech, Co., Ltd, Nanjing). Using CRISPR-mediated endogenous tagging, we introduced a C-terminal 3 × FLAG epitope into F1β (TGME49_261950), ASAP16 (TGME49_201800) and ASAP7 (TGME49_218940), TOM40(TGME49_218280) in the RH ΔKu80 line. The parasites were co-transfected with CRISPR/Cas9 plasmid and the 3Flag-CAT cassette prepared in the form of purified PCR products and selected with 20 μM chloramphenicol at 24 h after electroporation and recovery. Transfected parasites were cloned by limiting dilution and confirmed by PCR and IFA. Detailed information will be published elsewhere.

### Luciferase assays

1 × 10^6^ transgenic parasites expressing luciferase were inoculated into Vero cells in a 96-well plate. Parasites were treated with 10 μM BDQ for 2, 4, 8, 24, 48, 72, 96 and 120 h, then the drugs were removed and parasites were allowed to recover for 3 or 5 days. The cells were then harvested and the relative luminescence units (RLU) were detected by a fluorescence microplate reader (Tecan, Infinite M200 PRO, Männedorf, Switzerland) using the Bright-Lumi™ II Firefly Luciferase Assay Kit (Beyotime Biotech, Shanghai, China).

### RNA extraction and RNA-Seq

Fresh tachyzoites (3 × 10^7^) were inoculated into Vero cells and the noninvaded parasites were removed by several washes after 2 h. After 20 h of culture, the intracellular parasites were treated with 10 μM BDQ or vehicle control for an additional 12 h. Then, the treated parasites were collected for total RNA extraction using TRIzol regent (Invitrogen). The purity, concentration, and integrity of RNA were tested using the NanoPhotometer® (IMPLEN, CA, USA), the Qubit® RNA Assay Kit in the Qubit® 2.0 Fluorometer (Life Technologies, CA, USA) and the RNA Nano 6000 Assay Kit of the Bioanalyzer 2100 system (Agilent Technologies, CA, USA), respectively. Only qualified samples were used for library preparation. Illumina sequencing libraries were generated using the NEBNext® Ultra™ RNA Library Prep Kit for Illumina® (NEB, USA) according to the manufacturer’s recommendations. Sequencing was performed using the Illumina Novaseq 6000 platform, generating 276.9 million (46.1 million/sample) 150 bp paired-end reads. RNA-Seq analysis is based on three biological replicates per experimental group. The original sequencing data can be found in the Sequence Read Archive database under the accession number PRJNA951079.

### Bioinformatics

Paired-end clean reads were aligned to the *T. gondii* ME49 reference genome (ToxoDB, version 57) using TopHat2 [21]. Transcripts were assembled and counted by Cufflinks [22]. Differentially expressed genes (DEG) between BDQ- and DMSO-treated tachyzoites were calculated by the DEseq2 R package [23]. Gene ontology and KEGG pathway enrichment analysis was performed using topGO [24]. Gene expression with a fold change > 2 or < -2 and an adjusted *P*-value < 0.05 was defined as a significant differential expression.

### Statistical analyses

Statistical significance in plaque assay, invasion, proliferation, and parasite growth inhibition assay was evaluated by two-tailed unpaired *t*-tests, while the significance of survival was determined by log rank Mantel-Cox test using GraphPad Prism 9 (San Diego, CA, USA). Statistical data are expressed as the mean value ± standard deviation of data from at least three independent experiments.

## Results

### BDQ has a potent anti-*T. gondii* effect in vitro

**Figure 1:**
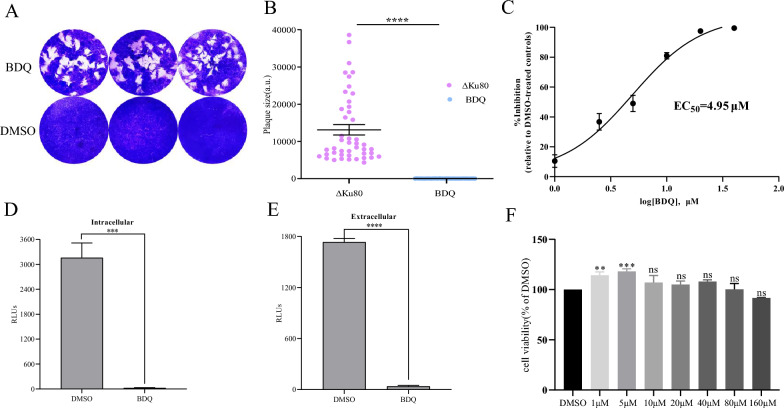
**Effectiveness of BDQ against** ***T. gondii.*** **A** Plaque assays for BDQ- or DMSO-treated parasites. HFF in each well were infected with 150 freshly harvested tachyzoites and incubated for 8 days undisturbed in the absence or presence of 10 μM BDQ. **B** Calculation of plaque area by measuring pixel points of randomly selected plaques (*n* > 30). Data were analyzed from three independent experiments. **C** Inhibition curve of BDQ against *T. gondii* in vitro. TgRH-Luc was treated with different concentrations of BDQ ranging from 0 to 20 μM, and relative luminescence units (RLU) were detected after 24 h. EC_50_ was calculated using the regression equation of log (inhibitor) vs. response-variable slope (four parameters) in GraphPad Prism 9 (San Diego, CA, USA). Means ± standard deviations (SD) are shown for the results of three independent experiments, each condition containing three replicates. **D** Effect of BDQ on intracellular parasites. Freshly (1 × 10^5^) released TgRH-Luc tachyzoites were allowed to invade the host cells for 2 h and were cultured for 24 h in medium containing BDQ (10 μM) or DMSO after several washes. Parasite growth was determined by the RLU. **E** The effect of BDQ on extracellular parasites. Fresh extracellular tachyzoites were pretreated with 10 μΜ BDQ or vehicle for 4 h, then inoculated into cells and cultured in drug-free medium for 24 h. **F** Cytotoxicity of BDQ on Vero cells. CCK-8 reagent was used to determine the cytotoxicity of different concentrations of BDQ on Vero cells. % viability = [(OD_BDQ_-OD_blank_) / (OD_DMSO_-OD_blank_)] × 100. Data are expressed as mean ± SD in triplicate. ns = non-significant, ** *P* < 0.01, *** *P* < 0.001, ****P < 0.0001.

The anti-*T. gondii* effect on BDQ was evaluated by plaque assay, which can fully reflect the growth of *T. gondii* in the entire lytic cycles. The results show no plaque formation after BDQ treatment, which was significantly different compared to the untreated group (*p* < 0.001) (Figures 1A and B).

To determine the effect of different concentrations of BDQ on tachyzoites, a drug inhibition assay was performed by using the luciferase-expressing *T. gondii* RH strain (TgRH-Luc). The results show that BDQ inhibited the growth of *T. gondii* tachyzoites in a dose-dependent manner, with the 50% effective concentration (EC_50_) of BDQ calculated to be 4.95 μM (95% confidence interval [CI], 3.27 to 6.25 μM) (Figures 1C). These indicate that BDQ has a potent anti-*T. gondii* effect.

Since BDQ treatment inhibited the ability of tachyzoites to establish infection (Figures 1A and B), the effect of BDQ on the intracellular and extracellular parasites was evaluated. Intracellular parasites show significant proliferation inhibition after 24 h of BDQ treatment compared to untreated parasites (Figure 1D). Extracellular TgRH-Luc tachyzoites were exposed to 10 μM BDQ for 4 h, and then the tachyzoites were washed and subsequently inoculated into the host cells for 24 h. The results show that the ability of tachyzoites to establish infection in the host cells was significantly inhibited even when BDQ was removed after 4 h (Figure 1E). These results suggest that BDQ significantly reduces the survival of intracellular and extracellular parasites.

To evaluate the toxicity of different concentrations of the compound on Vero cells, a Cell Counting Kit-8 (CCK-8) was used. Well-cultured cells in 96-well plates were treated with serial dilutions of BDQ (from 160 μM to 1 μM) for 24 h, and then the cell viability was determined by absorbance at 450 nm. The results show that BDQ was not toxic to Vero cells even at a very high level (160 μM), and the low BDQ concentration was found to have a promotive effect on the proliferation of Vero cells (Figure 1F). This experiment establishes a safe dose range for subsequent testing.

### BDQ rapidly kills* T. gondii* tachyzoites

**Figure 2:**
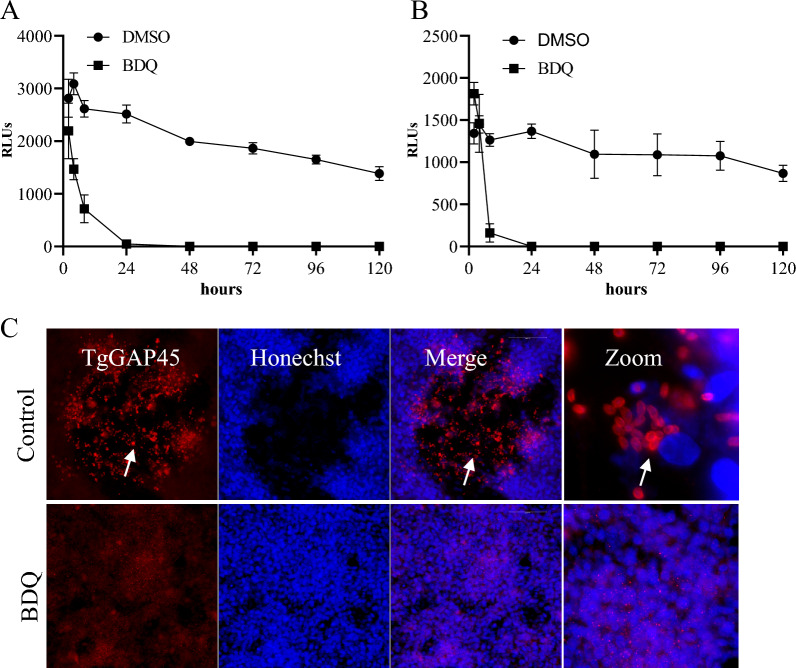
**BDQ rapidly kills** ***T. gondii*** **tachyzoites**. *T. gondii* tachyzoites expression firefly luciferase (TgRH-Luc) in HFF were exposed to 10 μM BDQ for 2, 4, 8, 24, 48, 72, 96 and 120 h, respectively. Then, the drugs were removed and parasites were allowed to recover for 3 **A** or 5 days **B**. The parasite numbers were presented by relative luminescence units (RLU). The experiments were performed with three independent replicates. **C** Parasite survival determined by immunofluorescent assay. Intracellular tachyzoites treated with BDQ or vehicle for 5 days, then the monolayers were crushed and the whole fragments were inoculated to new monolayers. The new monolayers were washed 5 h after inoculation and incubated for another 7 days. Then, parasites were fixed and stained by rabbit anti-GAP45 antibody and the nucleus was stained with Hoechst.

To assess the time course during which BDQ inhibits the growth of the parasites, *T. gondii* tachyzoites expressing firefly luciferase (TgRH-Luc) in HFF was exposed to 10 μM BDQ for 2, 4, 8, 24, 48, 72, 96 and 120 h, then the drugs were removed and parasites were allowed to recover for 3 (Figure 2A) or 5 days (Figure 2B). The growth of the parasites was represented by the relative luminescence units (RLU). The results show that parasite growth was near completely inhibited after 24 h of BDQ exposure (Figures 2A and B), and the treated parasite did not recover even after 5 days of further incubation (Figure 2B).

Additionally, intracellular tachyzoites were treated with BDQ or vehicle for 5 days, then the monolayers were crushed and the whole fragments were inoculated to new monolayers. We did not observe any parasites in the BDQ treated group even after resumption of growth for 7 days (Figure 2C), which reveals that the parasites were killed by the BDQ treatment.

### Disruption of parasite intracellular replication by BDQ

**Figure 3:**
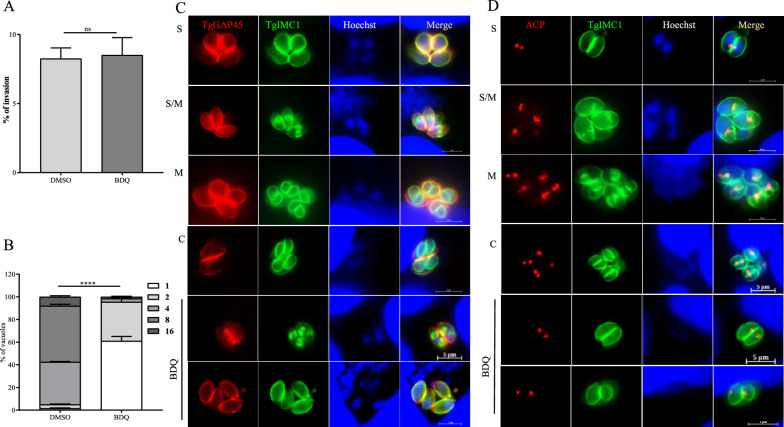
**Perturbation of parasite growth by BDQ**. **A** Comparison of the invasion ability of DMSO- and BDQ-treated parasites. Fresh tachyzoites invaded the host cell and were exposed to DMSO or BDQ (10 μM). The invasion ratio was calculated as the number of PV divided by the total number of host cells in one horizon (*n* = 30). Data are the mean ± SD (error bars) of three independent experiments. **B** The effects of BDQ on intracellular replication of the parasite. Cell cultured tachyzoites were treated with 10 μM BDQ or DMSO for 24 h. In each assay, a total of 100 PV of each group were counted. Experiments were compiled from three separate assays. Asterisks indicate statistical significance. **** *P* < 0.0001. **C**, **D** Immunofluorescence assay of IMC and apicoplast. Tachyzoites (3 × 10^5^) were used to invade HFF monolayers seeded on coverslips and then treated with DMSO or BDQ (10 µM) for 20 h, respectively. IFA was used to visually observe the changes in tachyzoites. The shapes of the parasites were labeled with rabbit anti-GAP45 antibody, and antibodies against TgIMC1 and TgACP were used to mark IMC and apicoplast subcellular organelles. The nucleus was stained with Hoechst (bar = 5 µm).

The plaque assay is a comprehensive assessment of the invasive nature, intracellular replication and egress capability of tachyzoites. The reduction in plaque formation could be caused by impairment at any of the steps in the lytic cycle. As a result, we assessed the invasion and replication process of the parasite. The results show no significant difference in the invasion rate of parasites treated with BDQ (10 µM) compared to DMSO-treated parasites (Figure 3A). Moreover, 60% of the PV treated with 10 μM BDQ contained only one tachyzoite, while the percentage of vacuoles containing 4 and 8 tachyzoites in the control group was 38.5% and 49.5%, respectively (Figure 3B).

To investigate the reason for replication arrest in BDQ-treated tachyzoite, progeny division was observed using IFA. The inner membrane complex (IMC) and apicoplast of tachyzoites were stained by IFA using specific antibodies. Compared to the control, there was no difference/change in the localization of IMC marker protein IMC1 and apicoplast marker protein ACP after BDQ treatment (Figures 3C and D). These results suggest that BDQ inhibits the proliferation of *T. gondii* tachyzoites, but probably not by altering the structure of the apicoplast and IMC.

### Influence of BDQ on the mitochondrial morphology of tachyzoites

**Figure 4:**
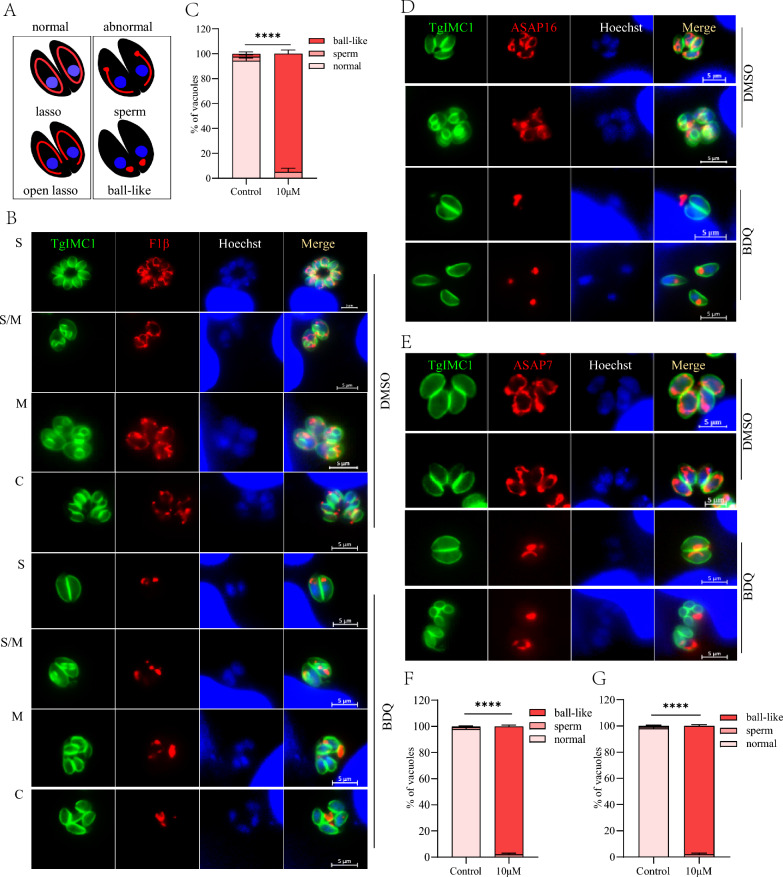
**Mitochondrial inner membrane defects due to BDQ treatment. A** Diagrams showing the different morphologies of *T. gondii* mitochondria. Mitochondrial morphology of intracellular parasites was observed by IFA. FLAG-labeled F1β **B** and **C**, ASAP16 **D** and APAP7 **E** parasite strains were treated with DMSO and 10 μM BDQ for 20 h and then observed after IFA using mouse anti-Flag and rabbit anti-TgIMC1 antibodies. Parasite shapes were observed with anti-TgIMC1 (green) and nuclear DNA was stained with Hoechst (blue). Scale bar = 5 μm. **C**, **F**, **G** Quantification of different types of mitochondrial morphology after 20 h of BDQ treatment using F1β- FLAG **C**, ASAP16-FLAG **F** and ASAP7-FLAG **G** strains. Quantification was performed based on 100 randomly selected tachyzoites from three independent experiments. Student *t*-test was used for statistical analysis (****P < 0.0001).

Previous reports have demonstrated that BDQ targets ATP synthase in *Mycobacterium* [14], so we further investigated whether ATP synthase was affected by BDQ treatment of *T. gondii*. Using CRISPR-mediated endogenous tagging, we introduced a C-terminal FLAG epitope into F1β, an inner mitochondrial ATP synthase marker in tachyzoites. Generally, the mitochondrion of intracellular parasites is distributed in the lasso formation around the nucleus, whereas extracellular parasites present sperm-like and collapsed mitochondrion [25] (Figure 4A). The immunofluorescence assay shows that F1β was distributed normally in the DMSO-treated parasite as previous reported [26] (Figure 4B). Interestingly, after BDQ treatment, F1β proteins were abnormally localized in 100% of tachyzoites and appeared as a lump with no visible mitochondrial profile (Figures 4B and C).

Since the mitochondrial ATP synthase complex subunit F1β is located at the mitochondrial inner membrane, the abnormal localization of F1β protein after BDQ treatment aroused our curiosity. To confirm that the abnormal localization of F1β is due to the direct effect of BDQ on F1β protein or its destruction of the mitochondrial inner membrane morphology, the localization of two other ATP synthase Fo subunits ASAP16 (TGME49_201800) and ASAP7 (TGME49_218940) of *T. gondii* was also observed by IFA [26]. C-terminal FLAG epitopes labeled as TgASAP16-FLAG and TgASAP7-FLAG parasites were constructed and treated with BDQ. In comparison with the untreated group, tachyzoites treated with BDQ also show abnormal localization of their ASAP16 and ASAP7 proteins, which were either crumpled or aggregated into irregular shapes instead of being distributed in a typical lasso format around the nucleus (Figures 4D–G). These results suggest that BDQ treatment leads to abnormal morphology of the mitochondrial inner membrane.

**Figure 5:**
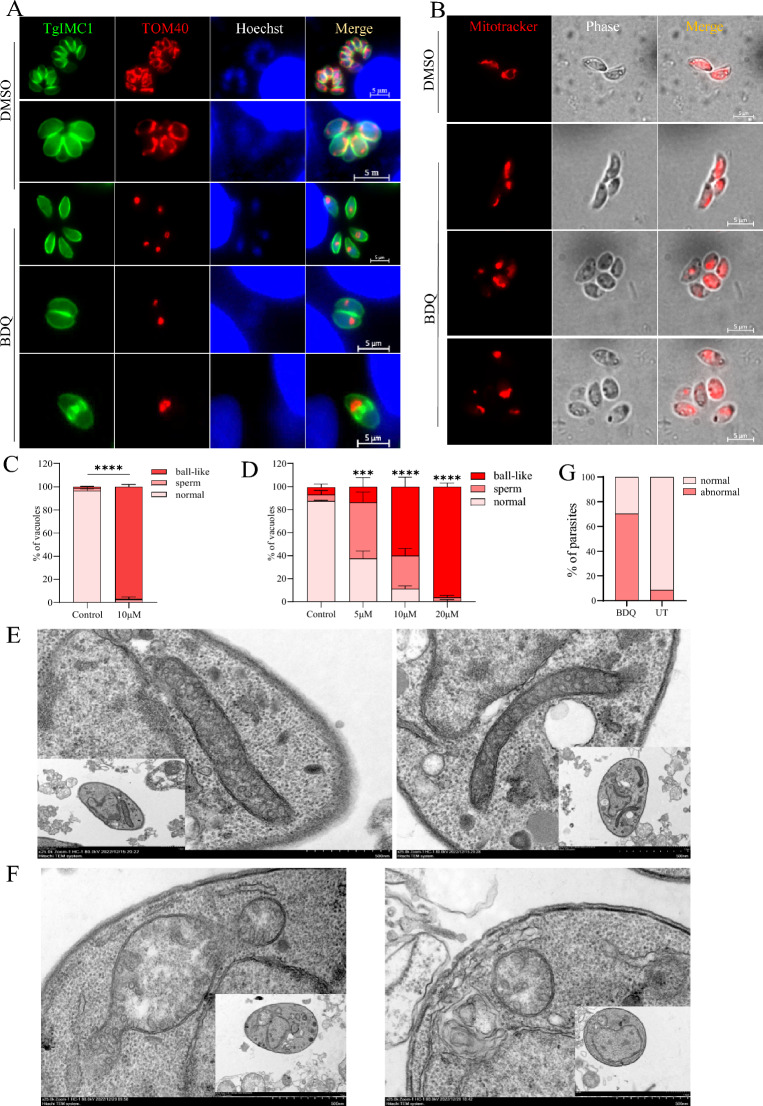
**Morphological changes of parasite mitochondrial outer membrane due to BDQ treatment. A** Immunofluorescence staining of *T. gondii* mitochondria outer membranes. Cell-cultured TgTOM40-FLAG parasites were treated with 10 μM BDQ and DMSO vehicle, and the localization of Tom40 protein was observed by IFA using anti-FLAG antibody (red). Parasite shapes were visualized by staining with anti-IMC1 antibody (green), and the nucleus was stained with Hoechst dye (blue). Scale bar = 5 μM. **B** Immunofluorescence staining of *T. gondii* mitochondria by Mito-tracker. *T. gondii* tachyzoites were co-cultured with different concentrations of BDQ (0—20 μM) for 20 h. Then, extracellular tachyzoites were stained with Mito-Tracker Red CMXRos. The different mitochondrial morphologies of BDQ-treated and untreated parasites were quantified by anti-FLAG antibody **C** and Mito-tracker staining **D** One hundred randomly selected tachyzoites were observed in each experiment. Statistical analysis was performed using two-way ANOVA with Tukey correction in Graph Pad Prism. * *P* < 0.05, ** *P* < 0.01 and ***P < 0.001. Transmission electron microscopy of *T. gondii* treated with DMEM (**E**) DMSO or BDQ (**F**). Enlarged images emphasis the mitochondria. (**G**) The abnormal mitochondria was statistically analyzed.

To confirm whether BDQ affects *T. gondii* mitochondrial outer membrane morphology, the mitochondrial outer membrane protein TOM40 was tagged (TgTOM40-FLAG) and used for immunofluorescence assay (Additional file 1). After treatment with BDQ, the parasite’s TOM40 was also found to be abnormally localized compared to the vehicle control. It was most likely collapsed into a ball-like structure, which was obviously different from its normal shape (Figure 5A). This phenotype was further confirmed by immunofluorescence staining of BDQ-treated and untreated parasites using Mito-tracker (Figure 5B). Our results show a significant increase in sperm and ball-like shaped mitochondria in BDQ-treated tachyzoites (Figures 5C, D). These results suggest that BDQ treatment can seriously alter the mitochondrial structure of *T. gondii*, which may be responsible for the parasite’s proliferation/growth defects.

The ultrastructure of *T. gondii* tachyzoites treated with BDQ was further analyzed by transmission electron microscopy. The mitochondrial membrane of the BDQ treated parasites show reduced density, and the inner matrix structure was partly destroyed (Figures 5E–G). These results suggest that BDQ impairs the parasite’s mitochondria outer membrane and inner structure integrity.

### BDQ affects the oxidative phosphorylation pathway gene transcription in *T. gondii*

**Figure 6:**
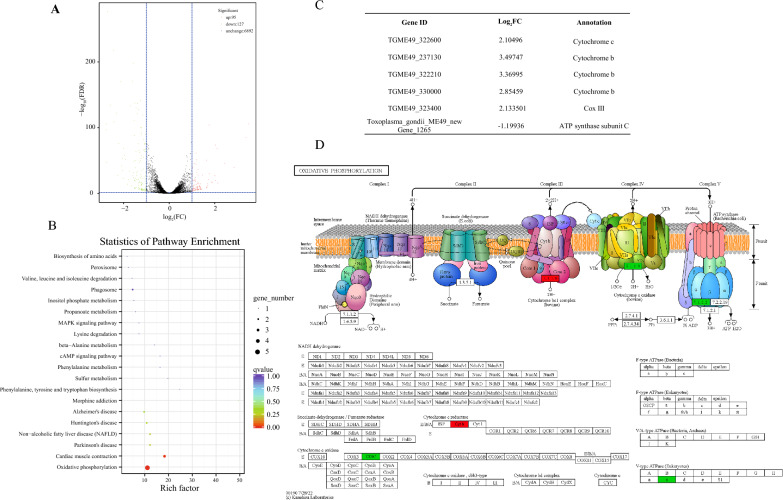
**Mitochondrial gene alterations in** ***T. gondii* due to BDQ treatment. A** Volcano plot showing the global *T. gondii* gene alteration after BDQ treatment*.* Significantly upregulated and downregulated genes are labeled in red and green, respectively. **B** KEGG pathway enrichment of DEG. The X-axis shows the rich factor and the Y-axis corresponds to the KEGG pathway. Dot size represents the number of DEG. The colors of the dots represent the adjusted *P*-values of the enrichment. **C** Five DEG related to the oxidative phosphorylation pathway. **D** KEGG pathway of oxidative phosphorylation.

To further explore the mechanism of mitochondrial damage caused by BDQ, we compared the gene expression patterns of BDQ-treated *T. gondii* treated by RNA-Seq. A total of 222 differentially expressed genes (DEG) were found after 12 h of 10 μM BDQ treatment (Additional file 2, fold change > 2, FDR < 0.05). The number of upregulated and downregulated genes in BDQ-treated tachyzoites was 95 and 127 (Figure 6A), respectively. KEGG pathway enrichment analysis revealed that DEG were significantly enriched in oxidative phosphorylation pathways (ko00190, q-value = 3.46E-05, Figure 6B). To explore the mechanism of action of BDQ on *T. gondii*, we further analyzed which oxidative phosphorylation pathway proteins were affected by BDQ treatment. The results show that the gene expression levels of proteins on mitochondrial respiratory chain complexes III, IV and V were significantly altered after treatment (Figures 6C, D). These results suggest that BDQ may interfere with the mitochondrial morphology and growth of *T. gondii* by affecting various proteins in the oxidative phosphorylation pathway. Additionally, we found that 28 *srs* (surface antigen glycoprotein 1-related sequences) genes were also down-regulated after BDQ treatment (Additional file 3). SRS proteins are believed to mediate attachment to host cells and activate host immunity to regulate parasite virulence [27]. However, the connection between BDQ treatment and *T. gondii srs* genes should be further explored.

### Mitochondrial dysfunction due to BDQ

**Figure 7:**
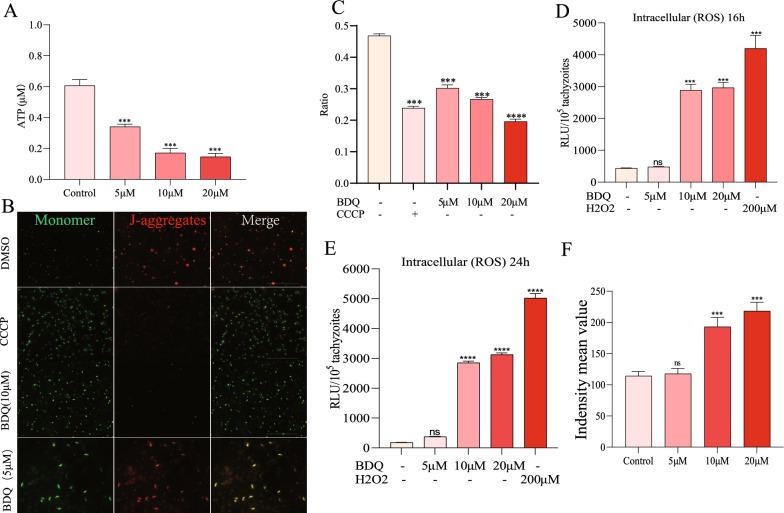
**Mitochondrial dysfunction and parasite autophagy due to BDQ treatment. A** Reduction of *T. gondii* ATP by BDQ treatment. Extracellular *T. gondii* tachyzoites (2 × 10^6^/per sample) were incubated with BDQ (0, 5, 10, or 20 μM) for 4 h at 37 °C. The tachyzoites were then lysed to determine ATP levels using a multi-label reader. **B** JC-10 probed fluorescence images of BDQ-treated *T. gondii* tachyzoites. Extracellular parasites (2 × 10^6^/per sample) were suspended with BDQ (0, 5, 10, or 20 μM) for 4 h at 37 °C and then detected using the JC-10 probe. DMSO and CCCP were used as negative and positive controls, respectively. J-Monomers (red) and J-aggregates (green) represent parasites with low and high mitochondrial membrane potentials, respectively. **C** Mitochondrial membrane potential in BDQ-treated *T. gondii* tachyzoites. The JC-10 probe fluorescence intensity ratio (RLU_J-aggregates_ / RLU_J-Monomers_) for each group was calculated. **D**, **E** Elevation of *T. gondii* ROS levels by BDQ. Tachyzoites (1 × 10^5^) were treated with BDQ for 16 h (D) or 24 h (E), and then ROS levels were detected using the DCFH-DA probe. **F** Parasite autophagy due to BDQ treatment. Intracellular *T. gondii* tachyzoites were treated with BDQ (0, 5, 10, or 20 μM) for 16 h at 37 °C, then the autophagic vacuoles of the parasites were stained with Monodansylcadaverine (MDC) for 30 min in the dark, and the mean value of fluorescence intensity was detected for each group. Data are expressed as means ± SD of three independent experiments. Ns: not significant, ***, *P* < 0.001, **** P < 0.0001.

BDQ treatment led to *T. gondii* replication arrest and alterations in mitochondrial morphology, and we speculated that the physiological function of mitochondria was affected. Considering that BDQ treatment mainly resulted in gene transcription and localization difference of the parasite mitochondrial ATP synthase complex subunits (Figures 5 and 6C, D), we first tested the ATP production of tachyzoites after BDQ treatment. The results show that the ATP levels of the parasites were significantly reduced after BDQ treatment in a dose-dependent manner (Figure 7A). The synthesis of ATP relies mainly on the driving process of proton motion. The mitochondrial membrane potential (ΔΨm) is an important component of proton motion, which is formed by protons pumped into the intermembrane space by the matrix.

Since ATP was significantly decreased after BDQ treatment, we speculated that the ΔΨm of the parasites might also be affected. As a result, ΔΨm in DBQ- or DMSO-treated parasites was further detected using the fluorescent probe JC-10. Carbonyl cyanide 3-chlorophenylhydrazone (CCCP), a potent mitochondrial oxidative phosphorylation uncoupling agent, was used as a positive control to drive the permeability of the inner mitochondrial membrane to H^+^, leading to a loss of membrane potential on both sides of the inner mitochondrial membrane. The results show that the ΔΨm of *T. gondii* was significantly reduced in a concentration-dependent manner after BDQ treatment (Figures 7B, C).

Defective or damaged mitochondria can lead to intracellular ROS accumulation and autophagic death in *T. gondii* [28, 29]. After BDQ treatment, various mitochondrial membrane proteins were located in abnormal spherical aggregation, suggesting that BDQ treatment may cause damage to the mitochondria of *T. gondii* and increase the ROS level. To verify this possibility, we examined the ROS levels of tachyzoites treated with BDQ. The results show that the ROS levels of tachyzoites increased significantly in a dose-dependent manner after 16 h of incubation (Figure 7D) and 24 h of BDQ (Figure 7E). To confirm BDQ-stimulated autophagy in *T. gondii*, autophagic vacuoles of the parasites were stained with Monodansylcadaverine (MDC) after incubation with BDQ. We found a significant elevation in the fluorescence intensity of *T. gondii* treated with 10 and 20 μM BDQ for 16 h, indicating that BDQ induces autophagy. In summary, BDQ treatment causes *T. gondii* mitochondrial dysfunction, which decreases ΔΨm and ATP and increases its ROS, ultimately possibly leading to autophagy in the parasites.

### BDQ protects mice from both acute and chronical infections

**Figure 8:**
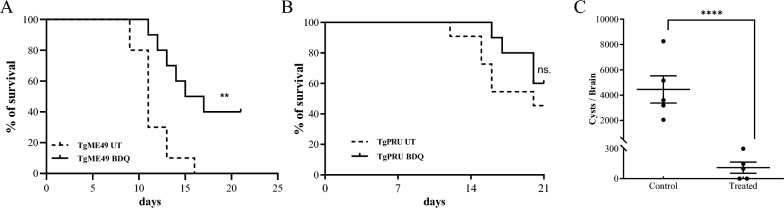
**BDQ protects mice from both acute and chronical infections.** Six-week-old female Kunming mice (*n* = 20) were infected intraperitoneally with 100 fresh ME49 **A** or 1000 Pru **B** tachyzoites, and were administrated with BDQ (50 mg/kg). The survival of mice was monitored for 21 days. **C** For the establishment of chronic infection, Kunming mice were infected (i.p.) with 500 Pru tachyzoites. After 21 days post-infection, mice (*n* = 5) were treated with BDQ (50 mg/kg) or vehicle for another 21 days. Then, the brain cysts were counted by FITC-labeled DBA staining. ** *P* value < 0.01; **** *P* value < 0.0001; ns: non-significant.

To address the treatment effect of BDQ on acute and chronical toxoplasmosis, the Kunming mice were infected with ME49 or Pru strain tachyzoites and administered with BDQ (50 mg/kg/day) or vehicle for 21 days. The BDQ provided significant protection to mice infected with the ME49 strain (*P* < 0.01) with a final survival rate of 30%, while all mice were dead 17 days post-infection in the untreated group (Figure 8A). However, no significant difference was observed for the survival rates in the Pru infected groups (Figure 8B).

The effectiveness of BDQ on the established chronic infection was also evaluated. Chronic toxoplasmosis in mice was established by infection with a sublethal dose of Pru strain tachyzoites for 21 days. The chronically infected mice were treated with BDQ (50 mg/kg/day) or vehicle for another 21 days, then the tissue cysts in brains were counted. Notably, a dramatic decrease (97.53%, *P* < 0.0001) was found in the chronical-infection model of Pru strain (Figure 8C). These results show that BDQ treatment significantly reduces the number of brain cysts in chronically infected mice.

## Discussion

*T. gondii* is a globally ubiquitous zoonotic apicomplexan parasite that can infect humans and almost all warm-blooded animals [30]. The limitations of currently available treatment options underscore the urgent need for better treatment options for acute and latent toxoplasmosis [31, 32]. Bedaquiline is a new diarylquinoline drug that has been approved by the U.S. Food and Drug Administration (FDA) for the treatment of tuberculosis [33]. It is believed to play an antibacterial role by inhibiting the activity of bacterial ATP synthase and interfering with bacterial energy production [14]. Notably, recent studies have suggested that BDQ may have anti-malaria activity [16]. In the present study, the anti-*T. gondii* activity of BDQ was investigated. Our results show that BDQ exerted a potent in vitro inhibitory effect on the growth of *T. gondii* in a dose-dependent manner, with the EC_50_ of BDQ towards this parasite being calculated to be 4.95 μM. We also show that BDQ inhibited intracellular replication of the parasite without affecting the invasion process of the parasites. More importantly, BDQ significantly reduces brain cysts for chronically infected mice. Furthermore, BDQ exhibited low cytotoxicity in Vero cells and, more importantly, it received conditional approval from the U.S. FDA in 2012 to be used in combination with other agents for the treatment of pulmonary MDR-TB at a dose of 400 mg once daily (QD) for 2 weeks, followed by 200 mg three times weekly (TIW) for 22 weeks [34]. Together, these results suggest that BDQ may be an excellent alternative for toxoplasmosis therapy.

Previous studies have shown that BDQ inhibits the ATP synthase of *M. tuberculosis* mitochondrial membranes by binding to subunit c [14, 15, 35]. However, BDQ is insensitive to human ATP synthase, which ensures that the target-based toxicity of BDQ and the possibility of interaction with human ATP synthase is extremely low [15, 35, 36]. Based on the mechanism of BDQ action in *Mycobacterium*, we presumed that BDQ may also affect subunits of the mitochondrial ATP synthase of *T. gondii*. By observing the localization of the F1β protein, a mitochondrial ATP synthase marker, we found that the protein was abnormally localized in a lump with no visible mitochondrial profile after BDQ treatment. Subsequently, we investigated the localization of the other two subunit proteins on the parasite ATP synthase and found that they both localized abnormally like F1β. Due to the abnormal and consistent localization of multiple proteins on the mitochondrial inner membrane, we speculated that BDQ may cause abnormal mitochondrial morphology throughout the parasite. To confirm this speculation, we observed the localization of the outer mitochondrial membrane marker TOM40 and found that TOM40 was abnormally localized, consistent with the localization of F1β. In addition, our results also observed that BDQ treatment did not affect the division and localization of apicoplast and inner membrane complex proteins. Therefore, these results demonstrate that BDQ can cause morphological abnormalities in tachyzoite mitochondria and eventually lead to replication arrest.

Considering the severe morphological abnormalities of mitochondria caused by BDQ treatment, we speculated that a variety of mitochondrial functions might be affected. RNA-Seq analysis shows that the mitochondrial oxidative phosphorylation pathway was affected in BDQ-treated parasites, mainly involving proteins on mitochondrial complexes III, IV and V. Usually, *Toxoplasma* oxidative phosphorylation occurs on a single mitochondrion [37–39]. ATP synthase is the key enzyme for ATP production by mitochondrial oxidative phosphorylation [40]. Our results show that the transcript levels of subunits above protein complex V were affected. Meanwhile, ATP test results show that ATP levels in *T. gondii* decreased in a dose-dependent manner after BDQ treatment, which is consistent with the transcriptome results. In addition, this is also consistent with previous results in *Mycobacterium* [41]. Therefore, we speculate that BDQ treatment may inhibit ATP synthesis by affecting the subunits on protein complex V.

ROS are by-products of aerobic metabolism, including superoxide anions, hydrogen peroxide and hydroxyl radicals, and play an important role in many biological processes [42]. Mitochondria are the main sites for the generation of ROS in cells, and the generation of ROS is closely related to the mitochondrial electron transport chain (ETC) [43, 44]. Mitochondrial proton leakage and electron leakage are coupled to oxidative phosphorylation and ROS production [43]. Electron leakage during electron transport leads to the production of mitochondrial ROS [43]. Among them, complex III has been described as an important source of ROS production in mitochondria [45, 46]. Our results show that the transcription levels of mitochondrial complex III proteins were significantly increased in *Toxoplasma* after treatment with BDQ. Meanwhile, BDQ also caused a significant increase in the level of ROS in *Toxoplasma*. These results suggest that the significant increase in BDQ-induced ROS in parasites may be due to altered transcription levels of genes on mitochondrial complex III in the oxidative phosphorylation pathway. Furthermore, ΔΨm is an important indicator of normal mitochondrial configuration and function. We found that an increase in ROS was accompanied by a decrease in ΔΨm and the occurrence of autophagy in BDQ-treated parasites. These indicators are also signs of impaired mitochondrial function.

Many anti-*Toxoplasma* drugs work by influencing mitochondrial function and inducing excessive ROS production. Previous studies have shown that treatment with *Moringa oleifera* led to increased ROS production and loss of mitochondrial membrane integrity, leading to the death of *T. gondii* [47]. Dihydroquinine disrupted tachyzoite mitochondrial membrane potential and ATP production, and elicited high ROS to kill *T. gondii* [48]. Therefore, we speculate that BDQ has a similar mechanism of action to that of *M. oleifera* and dihydroquinine. It damages the mitochondrial integrity and function of *T. gondii* by inducing excessive ROS production, blocking membrane potential formation and ATP synthesis, and ultimately leading to the death of the parasites.

In general, we report that BDQ leads to altered transcript levels of multiple complex subunits in the mitochondrial oxidative phosphorylation pathway, which causes structural abnormalities and dysfunction of mitochondria. It further leads to elevated levels of ROS in the parasite, which disrupts the mitochondrial membrane and subsequently reduces mitochondrial membrane potential and ATP synthesis. Finally, it leads to autophagy of the parasite cells. Our study investigated for the first time the in vitro anti-*T. gondii* effect of BDQ and demonstrates its mechanism of action, which provides a potential alternative for the treatment of toxoplasmosis.

### Supplementary Information

**Additional file 1: Strategy for endogenous 3 × FLAG tagging of the T. gondii TOM40 gene using the CRISPR/Cas9 system.**

**Additional file 2: Differently expressed T. gondii genes after bedaquiline treatment.**

**Additional file 3: Heat map showing differentially expressed srs genes after BDQ treatment.**

## Data Availability

The raw sequencing data generated in this study are publicly available in NCBI Sequence Read Archive under the accession number PRJNA951079.
