# Supplementary figures and images for: Mitochondrial dysfunction induced by bedaquiline as an anti-Toxoplasma alternative
Source: Vet Res. 2023 Dec 19;54:123. doi: 10.1186/s13567-023-01252-z (PMC10731829; doi:10.1186/s13567-023-01252-z)

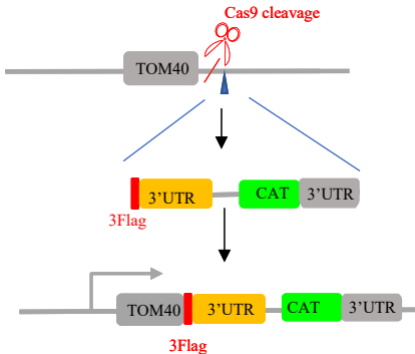

Supplement: Supplementary file 1 — Additional file 1: Strategy for endogenous 3 × FLAG tagging of the T. gondii TOM40 gene using the CRISPR/Cas9 system. [file 13567_2023_1252_MOESM1_ESM.pdf]

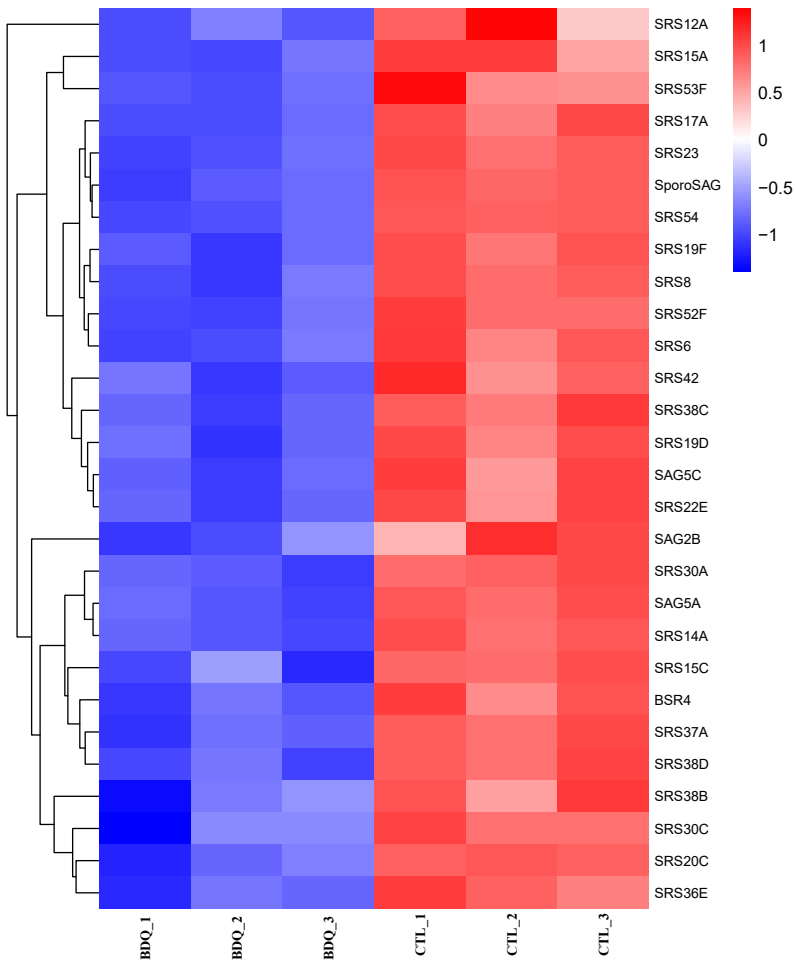

Supplement: Supplementary file 3 — Additional file 3: Heat map showing differentially expressed srs genes after BDQ treatment. [file 13567_2023_1252_MOESM3_ESM.pdf]
